# Budget impact analysis of ustekinumab in the management of moderate to severe psoriasis in Greece

**DOI:** 10.1186/1471-5945-12-10

**Published:** 2012-07-25

**Authors:** Georgia Avgerinou, Ioannis Bassukas, Georgios Chaidemenos, Andreas Katsampas, Marita Kosmadaki, Hara Kousoulakou, Athanasios Petridis, Brad Schenkel, Dimitrios Sotiriadis, Theofanis Spiliopoulos, Panagiotis Stavropoulos, Evgenia Toumpi, Loukas Xaplanteris

**Affiliations:** 1 Department of Skin and Venereal Diseases, University of Ioannina, Ioannina, Greece; 2 Department of Dermatology, Hospital for Skin and Venereal Diseases, Thessaloniki, Greece; 3 Department of Dermatology, University of Athens, Hospital “A. Syggros”, Athens, Greece; 4 Department of Dermatology, Medical School, Aristotle University of Thessaloniki, Thessaloniki, Greece; 5 Department of Dermatology, University of Patras, Patras, Greece; 6 Department of Dermatology and Venereology, “Attikon” General University Hospital, Athens, Greece; 7 Janssen Cilag Pharmaceutical SACI, Athens, Greece; 8 PRMA Consulting Ltd, Hampshire, UK; 9 Janssen Scientific Affairs, LLC, Horsham, PA, USA

## Abstract

**Background:**

The purpose of this study was to estimate the annual and per-patient budget impact of the treatment of moderate to severe psoriasis in Greece before and after the introduction of ustekinumab.

**Methods:**

A budget impact model was constructed from a national health system perspective to depict the clinical and economic aspects of psoriasis treatment over 5 years. The model included drug acquisition, monitoring, and administration costs for both the induction and maintenance years for patients in a treatment mix with etanercept, adalimumab, infliximab, with or without ustekinumab. It also considered the resource utilization for non-responders. Greek treatment patterns and resource utilization data were derived from 110 interviews with dermatologists conducted in February 2009 and evaluated by an expert panel of 18 key opinion leaders. Officially published sources were used to derive the unit costs. Costs of adverse events and indirect costs were excluded from the analysis. Treatment response was defined as the probability of achieving a PASI 50, PASI 75, or PASI 90 response, based on published clinical trial data.

**Results:**

The inclusion of ustekinumab in the biological treatment mix for moderate to severe psoriasis is predicted to lead to total per-patient savings of €443 and €900 in years 1 and 5 of its introduction, respectively. The cost savings were attributed to reduced administration costs, reduced hospitalizations for non-responders, and improved efficacy. These results were mainly driven by the low number of administrations required with ustekinumab over a 5 year treatment period (22 for ustekinumab, compared with 272 for etanercept, 131 for adalimumab, and 36 for infliximab).

**Conclusions:**

The inclusion of ustekinumab in the treatment of moderate to severe psoriasis in Greece is anticipated to have short- and long-term health and economic benefits, both on an annual and per-patient basis.

## Background

Psoriasis is a chronic, currently incurable, inflammatory skin disease. It is characterized by relapses and remissions, and is affected by several genetic and environmental factors [1]. Estimates of the worldwide prevalence of psoriasis range from 0.5% to 4.6% [2], with males and females being equally affected [1]. In Greece, the relative prevalence of psoriasis is 2.8% based on an 8-year prevalence study in an outpatient setting of a general state hospital dermatological teaching clinic [3]. Ethnic variations have been identified and Caucasians are more likely to suffer from the disease. The median age of onset is 28 years [2].

The most common type of psoriasis, occurring in more than 80% of cases, is plaque psoriasis or psoriasis vulgaris, characterized by well-demarcated erythematous scaly plaques [4]. Thirty-five percent of those with plaque psoriasis suffer from moderate to severe disease [5], which is usually defined as psoriasis affecting at least 10% of body surface area or a Psoriasis Area and Severity Index (PASI) score of 10 or more [1].

The chronic and incurable nature of plaque psoriasis indicates that it has a major social and economic impact on the community [6]. The psychological impact of psoriasis can be profound. The extent to which psoriasis affects a person’s health-related quality of life (HRQoL) is similar to that of other chronic diseases, such as arthritis, chronic lung disease, and type 2 diabetes [7]. Those with more severe psoriasis experience similar levels of anxiety to patients with conditions such as breast cancer, osteoporosis, or metastatic prostate cancer [8,9]. In a US study of 265 adults with psoriasis, 32% screened positive for depression and there was a graded relationship between depressive symptoms and HRQoL impairment (*P* < 0.001). More than 16% of those with high depression scores were treated with antidepressant medication. Both dissatisfaction with psoriasis treatment and illness-related stress were highly associated with depression [10]. Many people with psoriasis report moderate to severe feelings of stigmatization, anxiety, anger, and depression [11]. Increasing severity of psoriasis is closely correlated with suicidal ideation [12,13].

The annual, per-patient direct cost of psoriasis has been reported to be more than $14,600 in the US [14], £3,800 in the UK [15], and more than €5,000 in Italy [16]. The economic burden of psoriasis has not yet been evaluated in Greece.

One of the goals of psoriasis therapy is to reduce or clear plaques and induce remission [17]. The ideal therapy is an efficacious, long-lasting agent that is devoid of acute or long-term adverse effects, with minimal monitoring requirements and a dosing regimen that facilitates adherence [17]. These characteristics may help to reduce treatment costs while maintaining improvements in patients’ HRQoL [18,19].

Currently available systemic treatments for moderate to severe psoriasis include conventional drug therapies (cyclosporine, methotrexate, retinoids, and phototherapy) and biologics. The former have demonstrated varying degrees of efficacy, and long-term use can lead to serious side-effects [17]. In addition, systemic therapies lack durable efficacy (the symptoms of psoriasis recur shortly after withdrawal of conventional therapies) and have inconvenient administration schedules (e.g., daily dosing, multiple weekly exposures) [17].

On the other hand, the available biologic agents (infliximab, etanercept, adalimumab, and ustekinumab) provide specific, targeted regulation of the cells in the immune system and pathophysiologically designed intervention in the immunological disease cascade of psoriasis. They thus offer a treatment choice for patients who have moderate to severe disease where “conventional” systemic treatments have failed, are contraindicated, or not tolerated [20].

The primary safety concern with biologic agents is immunosuppression. Biologic agents are associated with increased risk of infection, serious infection and possibly malignancies [21]. The safety of biologic agents compared with conventional therapies for the treatment of psoriasis has not yet been precisely defined [22].

Ustekinumab is the most recent biologic agent to come to market. It was approved in Europe in January 2009 and has been shown to be well tolerated, to improve moderate to severe psoriasis, and to have a favorable administration and monitoring schedule (one subcutaneous injection every 12 weeks during the maintenance period) [23,24]. Moreover, in the Phase 3 randomized clinical trials, the PASI 75 results achieved with ustekinumab were sustained through at least 52 weeks [25,26].

The objective of this study was to estimate the annual and per-patient budget impact of the introduction of ustekinumab as a treatment alternative for patients with moderate to severe psoriasis in Greece, and to test the hypothesis that a treatment with improved risk–benefit and administration profiles compared with existing treatments can lead to cost savings.

## Methods

An economic model that estimated the annual and per-patient budget impact of ustekinumab was built in Excel 2007. The budget impact model estimated the impact of introducing ustekinumab into the treatment mix of biologic agents available for the treatment of moderate to severe plaque psoriasis in Greece, by comparing the costs incurred by the national health system before and after the introduction of ustekinumab.

All available biologic agents for the treatment of moderate to severe psoriasis in Greece, namely ustekinumab, etanercept, infliximab, and adalimumab, were included in the model as treatment options. Efalizumab was excluded from the analysis as its European marketing authorization was suspended in February 2009.

### Model structure

The economic analysis was performed from a national health system perspective. The model time frame was 5 years (base year 2009), during which the prevalence of psoriasis was assumed to be constant. The treatment response to biologic therapies was measured in terms of the probability of achieving a PASI 75 response, and the annual costs and resource utilization of both responders and non-responders to biologic treatment were considered in the model.

### Model inputs

#### Clinical data

Data on the clinical efficacy of biologic agents were taken from the meta-analysis conducted by Reich and colleagues [27]. This systematic literature review included all randomized controlled trials (until October 2008) that evaluated the efficacy of approved biologics for the treatment of moderate to severe psoriasis. A total of 20 studies enrolling 10,108 patients with psoriasis were included in the meta-analysis, including three Phase 3 trials of ustekinumab (PHOENIX 1, PHOENIX 2, and ACCEPT trials [25,26,28]). The estimated mean PASI 75 response rates per product are presented in Table 1.

**Table 1:** Mean percentage of patients achieving PASI 75 response with psoriasis biologic treatments

**Agent**	**Mean (%)**	**95% CI**
Adalimumab	58	49–68
Etanercept	52	45–59
Infliximab	80	70–87
Ustekinumab	69	62–75

^a^The relevant dosing scheme for each product is presented in Table 3.

CI, confidence interval; PASI, Psoriasis Area and Severity Index.

Source: Reich et al., 2012.

#### Resource utilization data

Data on medical resource use were collected through face-to-face interviews with dermatologists, the results of which were validated by an expert panel of 18 dermatologists.

The interviews were undertaken during January–March 2009 and included two stages. The first stage involved 5 minutes of computer-assisted telephone interviews (CATI), which aimed to identify dermatologists who were eligible for the second stage of the survey. CATI participants were randomly selected through a database including contact details for all registered members of the Hellenic Society of Dermatology and Venereology, which is publicly available on the official website of the society (http://www.edae.gr/). Randomization was based on market research techniques and resulted in a total of 200 dermatologists, who were both office and hospital based, and were from Athens, Thessaloniki (Salonica), and other urban centers.

Following CATI, a sample of 110 dermatologists was selected for the second stage of the primary research, based on specific quotas, the most important of which were the number of patients with psoriasis treated by each physician and the number of psoriasis patients for the treatment of whom the physician was personally responsible. The reason behind that was to include in the survey experienced dermatologists, actively involved in the treatment of psoriasis. The second stage included 30-minute face-to-face interviews with the 110 dermatologists, the characteristics of whom are presented in Table 2.

**Table 2:** Distribution of the 110 interviewed dermatologists by place of work and area

**Place of work**	**Number of dermatologists**	**%**
Office based	70	64
Hospital based	40	36
**Area**		
Athens	70	64
Thessaloniki	20	18
Other urban centers	20	18

A 40-item questionnaire (both quantitative and qualitative) was developed with the aim of exploring: a) epidemiologic data (number of patients with plaque psoriasis, percentage of patients with moderate to severe disease); b) treatment pathways (percentage of patients receiving pharmaceutical treatment, percentage receiving monotherapy versus combined treatment, percentage receiving biologic agents versus conventional systemic therapy); and c) resource utilization of both responders and non-responders (frequency and setting of administration, number of annual outpatient visits to physicians’ offices and hospitals, duration of hospitalizations) (see Additional fileAdditional file 1: Primary research questionnaire). Non-responders were defined as patients who did not achieve a PASI 75 response.

The interviews were based on a retrospective analysis on the use of biologic agents and on a hypothetical projection regarding the use of ustekinumab. In particular, the dermatologists were asked to consider their workload over the last month and provide information on moderate to severe psoriasis epidemiology and resource utilization based on their own experiences. The aim was to gather information on usual practice as opposed to best practice, as the former is more informative for determining the actual costs of treatment.

All data collected in the interviews were validated by an expert panel of 18 key opinion leaders in dermatology, who were selected on the basis of being either distinguished academics or managers of psoriasis treatment centers. The experts included in the panel represented all major geographic regions of Greece. No Ethics Committee approval was requested for the primary research component of the study, as the conduct of interviews with physicians and Experts' Panel are not subject to any approval according to the Greek legislation.

The questionnaire used during the expert panel procedure was the same as that used in the interviews. The findings from the original interviews were projected on a screen and the expert panel was asked to either confirm or reject them using a tele-voting system. If more than 60% of the experts disagreed with the findings of the fieldwork, they were invited to answer the same question based on their experience. The average of the experts’ answers was then included in the model. The experts’ opinions were also used to inform the model on the market shares of the biologic agents, with and without ustekinumab.

#### Cost data

Economic evaluations should go beyond the acquisition cost of drugs in order to reflect real-life clinical practice. Costing in this way requires that all resources used by a particular program or treatment are identified and valued.

To health economists, cost refers to the sacrifice of benefits made when a given resource is consumed in a program or treatment – in other words, the opportunity cost. The value of opportunity forgone in the next-best alternative use of health resources does not necessarily equate to the market price of the resources used, because the total costs of treatment should be considered. The total costs comprise the sum of all expenditures during a given time frame, including the direct costs incurred by the health care provider and patient, as well as the indirect costs to society of productivity lost. It is important to assess the relative importance of a cost item to the overall outcome, since the inclusion of minor costs may not be justified in certain cases. An example is when the cost of drug acquisition (a direct cost) far outweighs the magnitude of other costs, such as productivity loss (an indirect cost). Thus, in this model, only direct costs expressed in euros (2009) were considered.

Costs incurred during the maintenance years of therapy were not discounted. The costs associated with the management of adverse events were not incorporated in the model, as they were assumed to be the same across the biologics compared.

Drug acquisition costs were calculated on an annual basis for both the induction and maintenance years for each drug. The doses and number of administrations for each product were taken from the respective European Medicines Agency summaries of product characteristics (Table 3). According to expert medical opinion in Greece, for patients receiving ustekinumab, approximately 7% of patients weighing >100 kg receive 90 mg ustekinumab rather than the standard dose of 45 mg. This proportion was deemed to be too small to be incorporated in the model.

**Table 3:** Dosing scheme per product used in the analysis

**Agent**	**Dosing scheme**
Adalimumab	80 mg at week 0, 40 mg at week 1, then 40 mg every other week
Etanercept	50 mg twice weekly for 12 weeks, then 50 mg once weekly
Infliximab	5 mg/kg at weeks 0, 2, and 6, then every 8 weeks
Ustekinumab	45 mg at weeks 0 and 4, then every 12 weeks

Prices of the biologics were taken from officially published price bulletins from the Ministry of Development (http://www.ypan.gr). Tariffs from the largest social health insurance fund (http://www.ika.gr) were used to assess monitoring (outpatient visits to dermatologist), administration (visits to nurse, day hospital, or dermatologist’s private office), and inpatient costs.

In order to capture the total health care costs for the treatment of psoriasis, the type of costs considered in this analysis incorporated not only drug acquisition costs but also associated medical resource use costs, including resource costs for non-responders such as supportive care, outpatient visits, and hospitalization.

From a health care system perspective, office visits represent a substantial usage of health care infrastructure and are therefore an important component of fixed health care costs. For this reason, dispensing fees, administration costs, and office visits are commonly factored into economic analyses in the economic evaluation literature. This has been the preferred approach in numerous studies, even though the charges for administration costs may not necessarily differ between the therapies being compared (e.g., multivalent vaccines and infusion cancer therapies, in which office visits and administration costs are critical components of the economic analysis) [29,30]. This approach has been endorsed by the “Good Research Practices for Measuring Drug Costs in Cost Effectiveness Analyses” developed by the International Society for Pharmacoeconomics and Outcomes Research [31]. This document indicates that the administration cost of a medication is an integral part of the overall cost of treatment and, in fact, economic evaluations should go beyond the acquisition cost of drugs in order to reflect real-life clinical practice.

### Sensitivity analysis

In order to assess the impact of uncertainty of various model inputs on the results of the study, univariate sensitivity analyses were conducted on three variables that contributed to the cost of treatment and that were derived from the expert panel: hospitalization for non-responders; supportive care costs for non-responders; and market share of ustekinumab. Non-responders’ health care costs were varied, with the variation attributed to differences in resource use (i.e., the number of inpatient and outpatient visits). These costs were allowed to vary by ±10% in one-way sensitivity analyses, whereas the market share of ustekinumab for years 1–5 was allowed to vary by ±3% and ±6% (the latter being equally distributed to or withdrawn from all other biologic agents).

## Results

### Primary research results

Interviews with dermatologists and the expert panel validation that followed showed that 8% of patients with skin disease visiting a dermatologist are diagnosed with psoriasis. Of patients with psoriasis visiting a dermatologist, 63% have moderate to severe disease; of these, 28% are eligible for biologic therapies.

This primary research also showed that there is some variability in where patients receive their medicine, depending on the agent in question (Table 4).

**Table 4:** Administration setting (%) for biologic agents

**Agent**	**Hospital**	**Pharmacy**	**Dermatologist’s private office**	**Nurse at home**	**Alone at home**	**Health center/private clinic**
Adalimumab	9	12	2	8	60	8
Etanercept	11	12	4	9	58	7
Infliximab	99	0	1	0	0	0
Ustekinumab	68	3	2	16	12	0

Note: The percentages do not sum up to 100% across all rows because of rounding.

The number of visits of patients with psoriasis to health care professionals for monitoring the progress of their psoriasis, outside the visits for treatment administration purposes, is presented in Table 5. For all the biologics, patients more commonly visit hospitals than dermatologists’ private offices.

**Table 5:** Annual number of visits to health care professionals (excluding visits for administration)

**Agent**	**Hospital**	**Dermatologist’s private office**	**GP’s private office**
Adalimumab	6	5	2
Etanercept	6	5	2
Infliximab	5	2	0
Ustekinumab	6	4	1

The annual number of visits to dermatologists increases with disease severity (Table 6). Non-responders to biologic agents have on average 6 additional visits to hospitals and 6 additional visits to dermatologists per year compared with responders (Table 7).

**Table 6:** Mean annual number of visits to dermatologists, by severity of disease and work location of the dermatologist

**Work Location**	**Moderate psoriasis**	**Severe psoriasis**
Office-based dermatologist	7	8
Hospital-based dermatologist	6	10

**Table 7:** Mean annual number of additional visits for non-responders to biologic agents

**Visit Location**	**Mean number of additional visits**
Hospital	6
Dermatologist’s private office	6
GP private office	0

Finally, the expert panel provided estimates of the market share of ustekinumab and the other biologic agents, after the introduction of the former for the treatment of moderate to severe psoriasis (Table 8). Ustekinumab’s market share is expected to increase over a 5 year time horizon, starting from 9% in year 1 and reaching 26% in year 5.

**Table 8:** Estimated market share (%) of biologic agents for the treatment of moderate to severe psoriasis

**Agent**	**Base year**	**Year 1**	**Year 2**	**Year 3**	**Year 4**	**Year 5**
Adalimumab	30	27	25	24	23	23
Etanercept	45	42	39	37	36	35
Infliximab	25	22	20	18	17	16
Ustekinumab	0	9	16	21	24	26

### Model results

**Figure 1:**
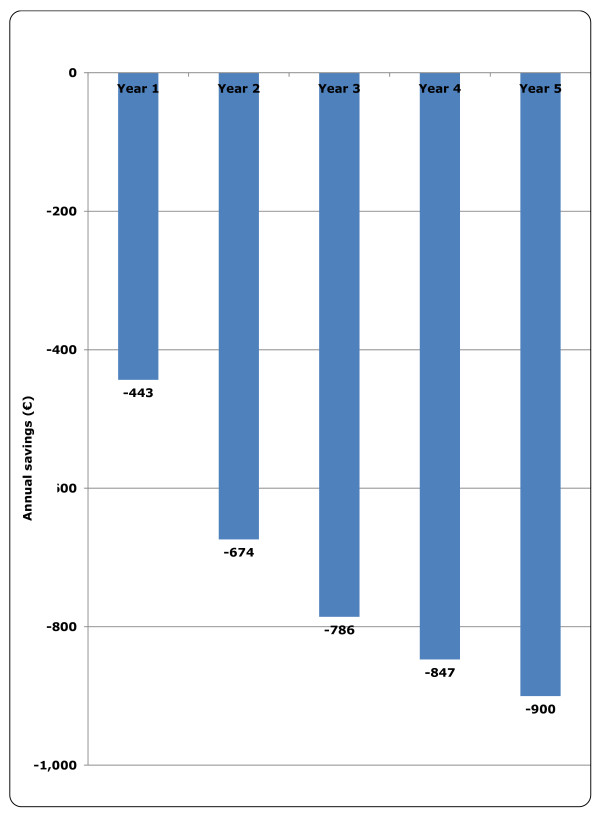
**Net budget impact of ustekinumab (annual cost savings per patient in €)**.

Based on the model results, the inclusion of ustekinumab in the biologic treatment setting for moderate to severe psoriasis is predicted to lead to total per-patient savings of €443 and €900 in years 1 and 5 of its introduction, respectively (Figure 1).

These results are mainly driven by the low number of administrations required with ustekinumab over a 5-year treatment period (22 for ustekinumab, compared with 272 for etanercept, 131 for adalimumab, and 36 for infliximab; Table 9). The cost savings for ustekinumab are also attributable to reduced hospitalization costs for non-responders and improved efficacy.

**Table 9:** Number of administrations for each product

**Agent**	**Induction year**	**Maintenance year**	**5 year total**
Adalimumab	27	26	131
Etanercept	64	52	272
Infliximab	8	7	36
Ustekinumab	5	4	22

### Sensitivity analyses

Results of the sensitivity analyses confirmed the robustness of the model to wide variation in inputs. Variation of non-responders’ outpatient costs by ±10% led to a minor change in the net budget impact of ustekinumab. Variation of non-responders’ hospitalization costs brought greater changes to ustekinumab’s budget impact, as hospitalization is an important cost driver of psoriasis. However, ustekinumab remained cost-saving at both ends of the range of inputs for hospitalization costs in the sensitivity analysis (Table 10).

**Table 10:** Sensitivity analysis of net budget impact of ustekinumab per patient (€)

	**Year 1**	**Year 2**	**Year 3**	**Year 4**	**Year 5**
Base case	−443	−674	−786	−847	−900
**Non-responders’ outpatient costs**					
−10%	−443	−673	−785	−846	−899
+10%	−444	−674	−786	−848	−901
**Non-responders’ hospitalization costs**					
−10%	−417	−647	−759	−820	−873
+10%	−470	−700	−812	−874	−927
**Ustekinumab share**					
−5%	−340	−559	−671	−732	−785
+5%	−547	−789	−901	−962	−1,015
−10%	−254	−441	−552	−614	−665
+10%	−648	−906	−1,018	−1,080	−1,133

Note: Negative values indicate budget savings.

In addition, even using the more conservative base-case market share assumption for ustekinumab (−10%), the introduction of ustekinumab as a treatment option for moderate to severe psoriasis is predicted to deliver substantial annual cost savings per patient, ranging from €254 in year 1 to €665 in year 5.

## Discussion

Moderate to severe psoriasis is a chronic, incurable disease, with substantial economic consequences for the health care budget. This is the first study to investigate the treatment patterns and resource utilization of psoriasis in Greece and the economic impact of the introduction of a new biologic treatment option.

The current study consisted of two parts: field work with questionnaires to dermatologists to identify resource use data; and a budget impact model to estimate the costs associated with adding ustekinumab to the current treatment options for psoriasis. The collection of resource use data through face-to-face interviews with physicians, rather than being derived from clinical trials or observational studies, could be criticized on the grounds of subjectivity and be considered a limitation of this study. However, in order to strengthen the validity of the data collected, an expert panel consisting of key opinion leaders was set up to assess the primary results.

The selection of dermatologists to participate in the primary research was mainly based on the level of experience they had with psoriatic patients, the rationale being that physicians with more experience on psoriasis would be able to provide more robust estimates for the parameters investigated in the study. As a result, the estimated eligible patient population entering the model in year 1 is potentially shifted upwards compared to actual numbers, leading to a subsequent overestimate in the budget impact of the related biologic treatments. However, the results of the present study in terms of cost differences across treatments, are not affected, as the eligible population is the same for all treatments and therefore has a proportionate impact on respective budgets.

The results reveal that etanercept is currently the preferred treatment option for moderate to severe psoriasis, followed by adalimumab and infliximab. An interesting finding is that although etanercept and adalimumab are administered at home for the majority of patients, patients more commonly visit hospital-based physicians than the private offices of dermatologists to monitor their treatment progress. This may be attributed to the fact that specialized psoriasis centers are located in some hospitals.

The results also show that resource utilization and related costs increase with disease severity, a finding confirmed by the literature [32]. Moreover, the investigation of the budget impact of adding ustekinumab as a treatment option for psoriasis shows that this would lead to substantial cost savings, even in the first year of its introduction.

The therapeutic benefits of ustekinumab have been confirmed in three large Phase 3 trials in patients with moderate to severe psoriasis [25,26,28]. These studies found that a significantly higher proportion of patients receiving ustekinumab compared with placebo or etanercept achieved PASI 75 at 12 weeks. Other efficacy measures, including the Physician’s Global Assessment at week 12, also favored ustekinumab [25,26,28]. Moreover, subcutaneous ustekinumab was generally well tolerated [24-26,28]. Treatment with ustekinumab has also been found to result in significantly improved HRQoL (Dermatology Life Quality Index) [33,34], lowered depression and anxiety rates based on the Hospital Anxiety and Depression Scale [34], and improved employability and productivity [35].

A possible shortcoming of the present study is that hospitalization and outpatient costs may have been underestimated. Social health insurance fund tariffs, which have been used in this model, do not reflect actual costs; actual costs are higher than the amount reimbursed by insurance funds.

Another limitation is that indirect costs were not considered. Indirect costs related to psoriasis include lost work time (i.e., days missed from work) and reduced productivity. Indirect costs increase with disease severity and can be significant [32]. In a UK study, 59.3% of patients with psoriasis who were still working had lost an average of 26 days from work in the previous year because of their psoriasis, and of the 180 patients not working, 33.9% reported not working because of their psoriasis [36]. A study in Germany showed that the mean indirect costs and loss of productivity per patient with psoriasis were €1,310 per year, accounting for 19.5% of total psoriasis costs [37]. However, clinical trials of biologics, including ustekinumab, demonstrate that patients who respond to treatment experience improvements in productivity and reductions in work-day loss. Therefore, the omission of indirect costs in this analysis is unlikely to adversely affect the research findings.

An important finding of this study is that, based on expert opinion, 67.5% of ustekinumab-treated patients will initially be administered the product in hospital rather than at home or in their dermatologist’s private office. This is probably due to physicians’ reservations regarding a new biologic agent. According to the expert panel, reinforcement of ustekinumab’s efficacy and safety data with local dermatologists’ own experience is likely to lead to patients receiving the drug outside of the hospital setting. The expert panel’s opinion was that similar treatment patterns as with etanercept and adalimumab (where 58% and 60% of patients, respectively, perform administration at home) are expected for ustekinumab users in the future.

Two Phase 3 studies of ustekinumab have shown that the drug has a comparable safety profile with self-administration versus administration by a health care professional [25,26]. A movement toward more frequent administration at home rather than in the hospital setting could further reduce the direct costs of ustekinumab use.

Overall, the present study investigated, for the first time in Greece, the treatment patterns and resource utilization of patients with moderate to severe psoriasis. These findings may be used to inform the development of national treatment guidelines in psoriasis and health policy resource allocation decisions.

## Conclusions

Ustekinumab offers a promising alternative to currently approved biologic agents for psoriasis, with both short- and long-term economic benefits. Based on the present model calculation, the introduction of ustekinumab as an alternative treatment option for moderate to severe psoriasis in Greece is anticipated to bring substantial cost savings to the national health care budget.

## Competing interests

HK has provided consultancy services to Janssen-Cilag Greece Pharmaceutical SACI. LX was a paid employee of Janssen-Cilag Greece Pharmaceutical SACI until December 2009. BS is a paid employee of Janssen Scientific Affairs, LLC, Horsham, PA, USA. IB, GC, AK, MK, AP, DS, TS, PS, and ET received honoraria from Janssen-Cilag Greece Pharmaceutical SACI for their participation in the expert panel.

## Authors’ contributions

HK and LX participated in the design and coordination of the study and the analysis and interpretation of the data, and drafted the manuscript. BS revised the manuscript critically for important intellectual content. GA, IB, GC, AK, MK, AP, DS, TS, PS and ET participated in the expert panel for the validation of the data on medical resource utilization and contributed to the manuscript preparation. All authors read and approved the final manuscript.

## Pre-publication history

The pre-publication history for this paper can be accessed here:

http://www.biomedcentral.com/1471-5945/12/10/prepub

## Supplementary Material

Additional file 1: **Primary research questionnaire.** This is the full 40-item questionnaire which was used both during the face-to-face interviews with the dermatologists and during the expert panel. This is a pdf document and can be viewed with Adobe Acrobat. (PDF 78 kb) Click here for file
