# Supplementary material for: Budget impact analysis of ustekinumab in the management of moderate to severe psoriasis in Greece
Source: BMC Dermatol. 2012 Jul 25;12:10. doi: 10.1186/1471-5945-12-10 (PMC3420305; doi:10.1186/1471-5945-12-10)
Supplement: Additional file 1: — Primary research questionnaire. This is the full 40-item questionnaire which was used both during the face-to-face interviews with the dermatologists and during the expert panel. This is a pdf document and can be viewed with Adobe Acrobat. (PDF 78 kb) [file 1471-5945-12-10-S1.pdf]

Additional file 1: Primary research questionnaire

This questionnaire was used both during the face-to-face interviews with the dermatologists and during the expert panel

1. During the last month, how many patients did you see in total? (please report the number of patients with any condition that visited you –please count once, even if you saw a patient twice during the month)
2. Of the patients that you saw during the last month, how many had psoriasis?
3. Of the patients with psoriasis that you saw during the last month, how many had
    a. psoriasis and psoriatic arthritis
    b. plaque psoriasis only
    c. other type of psoriasis only
4. Of the patients with plaque psoriasis that you saw during the last month, how many:
    a. got diagnosed with the disease for the first time?
    b. were previously diagnosed psoriatic patients?
5. Of the newly diagnosed patients with psoriasis that you saw during the last month, how many:
    a. came directly to you?
    b. were referred by another specialty?
    c. were referred by another non medical specialty?
6. Of the newly diagnosed patients with psoriasis that were referred by a medical specialty, how many:
    a. came by being diagnosed with psoriasis

    b. came by being diagnosed with another disease
    c. came without a diagnosis
7. Of the patients with plaque psoriasis that you saw during the last month, how many:
    a. did you personally treat without referring them to another doctor?
    b. did you refer to another doctor for their treatment?
8. Of the patients with plaque psoriasis that you personally treated during the last month, how many had:
    a. Mild psoriasis
    b. Moderate psoriasis
    c. Severe psoriasis
9. On average, how many times per year:
    a. does a patient with mild psoriasis visit you?
    b. does a patient with moderate psoriasis visit you?
    c. does a patient with severe psoriasis visit you?
10. Of the patient with moderate to severe plaque psoriasis that you personally treated, how many:
    a. received pharmaceutical treatment?
    b. received non-pharmaceutical treatment?
    c. did not receive any treatment?
11. Of the patients with moderate to severe plaque psoriasis that you personally treated with pharmaceutical treatment, how many:
    a. received monotherapy?
    b. received combined treatment?
12. Of the patients with moderate to severe plaque psoriasis that received monotherapy, how many were treated with:

    a. biologic agents?
    b. conventional systemic therapy?
13. Of the psoriatic patients that you treat with biologic agents, how many weighted more than 100 kg?
14. What percentage of the moderate to severe psoriatic patients that you treated with biologic agents over the last month received:
    a. Etanercept
    b. Adalimumab
    c. Infliximab

    (Note: all percentages should sum to 100%)
15. Of the patients with moderate to severe plaque psoriasis that you prescribed Etanercept, to how many did you:
    a. give it as initial treatment
    b. follow up without change in the treatment
    c. changed the treatment scheme (e.g. adjust dose or time intervals)
16. Of the patients with moderate to severe psoriasis that you prescribed Adalimumab, to how many did you
    a. give it as initial treatment
    b. follow up without change in the treatment
    c. changed the treatment scheme (e.g. adjust dose or time intervals)
17. Of the patients with moderate to severe psoriasis that you prescribed Infliximab, to how many did you
    a. give it as initial treatment
    b. follow up without change in the treatment
    c. changed the treatment scheme (e.g. adjust dose or time intervals)

18. What dosing scheme for Etanercept did you use in the moderate to severe psoriatic patients that you treated during the last month?
19. What dosing scheme for Adalimumab did you use in the moderate to severe psoriatic patients that you treated during the last month?
20. What dosing scheme for Infliximab did you use in the moderate to severe psoriatic patients that you treated during the last month?
21. What is the average duration of treatment in psoriatic patients for each of the biologic agents listed below (please report in months):
    a. Etanercept
    b. Adalimumab
    c. Infliximab
22. Out of 100 patients with moderate to severe psoriasis who receive Etanercept, how many have it administered:
    a. in a hospital setting
    b. in a retail pharmacy
    c. at the dermatologist's private office
    d. at home by a nurse
    e. at home, alone
    f. at health centres/ private clinic

    (Note: all percentages should sum to 100%)
23. Out of 100 patients with moderate to severe psoriasis who receive Adalimumab, how many have it administered:
    a. in a hospital setting
    b. in a retail pharmacy
    c. at the dermatologist's private office
    d. at home by a nurse

   e. at home, alone
   f. at health centres/ private clinic

   (Note: all percentages should sum to 100%)

24. Out of 100 patients with moderate to severer psoriasis who receive Infliximab, how many have it administered:
   a. in a hospital setting
   b. in a retail pharmacy
   c. at the dermatologist's private office
   d. at home, by a nurse
   e. at home, alone
   f. in a health center/ private clinic

   (Note: all percentages should sum to 100%)

25. Out of 100 patients with moderate to severer psoriasis who would receive Ustekinumab, how many would you expect to have it administered:
   a. in a hospital setting
   b. in a retail pharmacy
   c. at the dermatologist's private office
   d. at home, by a nurse
   e. at home, alone
   f. in a health center/ private clinic

   (Note: all percentages should sum to 100%)

26. On average, how many times per year does a psoriatic patient receiving Etanercept visit a health professional (excluding visits for administration):
   a. in a hospital setting?
   b. at the dermatologist's private office?
   c. at the GP's private office?

27. On average, how many times per year does a psoriatic patient receiving Adalimumab visit a health professional (excluding visits for administration):
    a. in a hospital setting?
    b. at the dermatologist's private office?
    c. at the GP's private office?
28. On average, how many times per year does a psoriatic patient receiving Infliximab visit a health professional (excluding visits for administration):
    a. in a hospital setting?
    b. at the dermatologist's private office?
    c. at the GP's private office?
29. On average, how many times per year would you expect a psoriatic patient receiving Ustekinumab to visit a health professional (excluding visits for administration):
    a. in a hospital setting?
    b. at the dermatologist's private office?
    c. at the GP's private office?
30. What is the percentage of patients with moderate to severe plaque psoriasis receiving a biologic agent that are hospitalised over a year, for a reason relating to their treatment?
31. For the moderate to severe psoriatic patients that are hospitalised, what is the average length of stay? (please report in days)
32. What percentage of patients receiving Etanercept does not respond to treatment?
33. What percentage of the patients receiving Adalimumab does not respond to treatment?

34. What percentage of the patients receiving Infliximab does not respond to treatment?
35. A moderate to severe psoriatic patient who does not respond to a treatment with a biologic agent, how many additional times on average does he have to visit a health professional per year?
36. What percentage of the moderate to severe psoriatic patients who do not respond to a treatment with a biologic agent are hospitalised over a year?
37. For the non-responders that are hospitalised, what is the average length of stay? (please report in days)
38. Of the moderate to severe psoriatic patients receiving Etanercept, how many would you switch to ustekinumab?
39. Of the moderate to severe psoriatic patients receiving Adalimumab, how many would you switch to ustekinumab?
40. Of the moderate to severe psoriatic patients receiving Infliximab, how many would you switch to ustekinumab?
